# What happened to health service utilization, health care expenditures, and quality of care in patients with acute pancreatitis after implementation of global budgeting in Taiwan?

**DOI:** 10.1097/MD.0000000000012620

**Published:** 2018-10-12

**Authors:** Ya-Lin Ko, Jyun-Wei Wang, Hui-Mei Hsu, Chia-Hung Kao, Chun-Yi Lin

**Affiliations:** a Division of Allergy, Immunology, and Rheumatology, Department of Internal Medicine, Show Chwan Memorial Hospital; b Division of Gastroenterology, Department of Internal Medicine, Chang Bing Show Chwan Memorial Hospital; c Department of Management, Show Chwan Memorial Hospital; d Department of Management, Chang Bing Show Chwan Memorial Hospital; e Graduate Institute of Clinical Medical Science and School of Medicine, College of Medicine, China Medical University; f School of Medicine, College of Medicine, Fu Jen Catholic University; g Department of Nuclear Medicine, Show Chwan Memorial Hospital; h Department of Nuclear Medicine, Chang Bing Show Chwan Memorial Hospital, Taiwan.

**Keywords:** acute pancreatitis, global budgeting system, health care expenditures, health service utilization

## Abstract

**Aim:**

Acute pancreatitis is associated with significant morbidity and mortality. In the United States, more than 3,00,000 patients are admitted and about 20,000 die from acute pancreatitis per year. In Taiwan, the incidence rate of acute pancreatitis is 0.03% and the mortality rate among severe acute pancreatitis is 16.3%. The aim of the study was to evaluate the impact of the global budgeting system on health service utilization, health care expenditures, and quality of care among patients with acute pancreatitis in Taiwan.

**Materials and methods:**

The National Health Insurance Research Database (NHIRD) was used for analysis. Data on patients with acute pancreatitis diagnosed during the period 2000 and 2001 were used as baseline data, and data from 2004 and 2005 were used as post-intervention data. The length of stay (LOS), diagnostic costs, drug cost, therapy costs, total costs, risk of readmission within 14 days, and risk of revisiting the emergency department (ED) within 3 days of discharge before and after implementation of the global budgeting system were compared and analyzed.

**Results:**

Data on 2810 patients with acute pancreatitis were analyzed in this study. There was a significant difference in mean LOS before and after introduction of the global budget system (7.34 ± 0.22 days and 7.82 ± 0.22 days, respectively; *P* < .001)). The mean total costs before and after implementation of the global budget system were Taiwan dollars (NT$) 28,290.66 ± 1576.32 and NT$ 42,341.83 ± 2285.23, respectively. The mean rate of revisiting the ED within 3 days decreased from 9.9 ± 0.9% before adoption of global budgeting to 7.2 ± 0.6% after implementation of the system. The mean 14-day re-admission rates before and after introduction of global budgeting were 11.6 ± 1.0% and 7.9 ± 0.7%, respectively.

**Conclusion:**

The global budget system was associated with significantly longer length of stay, higher health care expenditures, and better quality of care in patients treated for acute pancreatitis.

## 1 Introduction

Acute pancreatitis is a common disease and is associated with significant morbidity and mortality. Although it has a mild and self-liming course, it can be severe and cause complications that carry a significant risk of death. In the United States, more than 3,00,000 patients are admitted with incident pancreatitis and about 20,000 die from the disease each year.^[1–3]^ In Taiwan, the incidence rate of acute pancreatitis is 0.03% and the mortality rate among severe acute pancreatitis is 16.3%.^[4]^ The pancreas is a source of hormones, insulin, and glucagons that are important for regulating metabolism. The key exocrine function of the pancreas is to produce pancreatic juices that contain water, bicarbonate, and various digestive enzymes.^[5]^

There are 2 types of acute pancreatitis, interstitial (about 80% of cases, with a 5%–10% mortality rate) and necrotizing (about 20% of cases, with a 50% mortality rate). The leading causes of pancreatitis are gallstones, which are responsible for about 40% of cases, and alcoholism, which is responsible for about 35% of cases.^[6–10]^ It has been found that only 5% to 10% of patients with alcoholism develop acute pancreatitis, which argues that genetic or environmental cofactors are important in the development of alcoholic pancreatitis. Smoking is another important cofactor.^[11–15]^

Patients who present with 2 of the following 3 manifestations are diagnosed as having acute pancreatitis: characteristic upper abdominal pain, elevated levels of pancreatic enzymes, and findings of ultrasonography (US), computed tomography (CT) or magnetic resonance imaging (MRI) suggesting acute pancreatitis. Epigastric pain radiating to the back, nausea, and vomiting are the main symptoms of acute pancreatitis. Abdominal pain, which occurs in about 95% of cases, typically occurs acutely, and rapidly reaches maximum intensity.^[1,16]^ Two key diagnostic tests for acute pancreatitis involve measuring the levels of serum amylase and serum lipase. Measurement of serum amylase is a very sensitive diagnostic test, but has low specificity.^[17]^ Measurement of serum lipase, however, has a much higher sensitivity (90%) and specificity (90%) for diagnosing acute pancreatitis.^[18]^ Treatment for acute pancreatitis includes pain control, intravenous fluids, pancreatic rest, and monitoring for complications. Aggressive surgical intervention may be necessary for patients who have signs of infected necrosis and whose disease is not controllable by conservative methods.^[19]^ Nutrition should be instituted within a few days.

The National Health Insurance (NHI) program is the backbone of the health care system in Taiwan. The NHI is the major source of health financing and covers 99% of the population. National health care expenditure in Taiwan increased from 5.3% to 6.0% of gross national product during the period 1995 to 2001. The NHI used to operate on a fee-for-service (FFS) basis and as a result health care spending increased by approximately 50% during the period 1995 to 2001. In order to prevent unlimited and rapid growth of spending on health care, the Bureau of National Health Insurance (BNHI) implemented global budgeting to modify the FFS mechanism in 2002. Global budgeting in Taiwan is an overall spending target, designed to limit the volume of service and its total price.^[20–24]^ The global budgeting program adopted in Taiwan is an expenditure-cap system with a “floating point value” mechanism. The national budget for a given year is determined before the end of the previous year by consultation between the BNHI and hospital representatives. The aggregate level of expenditure in the previous year and age structure of the population determine the size of the national budget. The monetary value of each point has been equal to the fixed budget divided by the number of points that all hospitals claim, so it fluctuates to precisely match the pre-determined budget. Reimbursement to providers is based on an existing FFS schedule, which lists a relative value or number of points for each item of service. The degree of cuts in reimbursement was measured based on the quarterly monetary value of each point.

The objectives of this study were to compare the health service utilization (length of hospital stay), health care expenditures (eg, total costs, drug costs, costs related to diagnoses, and costs pertaining to treatment), and the quality of care (eg, risk of readmission within 14 days and risk of revisiting the emergency department (ED) within 3 days) before and after adoption of the global budgeting system.

## 2 Materials and methods

This was a pre-post comparison study. The study was based in part on data from the National Health Insurance Research Database (NHIRD) provided by the BNHI, Department of Health and managed by the National Health Research Institutes. The database contains registration files and original claims data for reimbursement. The NHIRD is provided to scientists in Taiwan for research purposes. Each year, the BNHI collects data from the National Health Insurance program and sorts it into data files. These data files are de-identified by scrambling the identification codes of both patients and medical facilities and then sent to the National Health Research Institutes to form the original files contained in the NHIRD.^[25]^

Based on the registration files and original claims data in the NHIRD, specific data subsets can be re-constructed for research purposes. The registration datasets, systemic sampling inpatient expenditures by admissions (DD file) and systemic sampling ambulatory care expenditures by visits (CD file) were used in this study. The interpretation and conclusions contained herein do not represent those of the Bureau of National Health Insurance, the Department of Health, or the National Health Research Institutes.^[25]^

### 2.1 Systemic sampling CD and DD files

Approximately, 0.2% of the ambulatory care expenditures, by visit, CD file extracted by systematic sampling method on a monthly basis, together with the related records in details of ambulatory care orders form the systematic sampling CD file; 5% of the inpatient expenditures, by admission, DD file extracted by systematic sampling method on a monthly basis, together with the related records in details of inpatient orders form the systematic sampling DD file.^[25]^ A total of 1 million cases of systemic sampling dataset were used in this study.

### 2.2 Study population

Subjects in systemic sampling CD and systemic sampling DD files with a clinical diagnosis of acute pancreatitis, ICD-9 code 577.0, in 2000, 2001, 2004, and 2005 were recruited for comparison and analysis. ICD-9 577.0 was used to define the disease in both inpatient and ambulatory codes. One time ICD-9 coding was used to be scrutinized before they can define the acute pancreatitis. The definition of readmission was that readmission event for the treatment of acute pancreatitis. The admission and readmission events need the same ICD-9 coding.

The global budget system in Taiwan was fully implemented in 2002; therefore, data from 2000 and 2001 were used as baseline data (pre global budget). Data from 2004 and 2005 were used as post intervention data (post global budget). In 2003, there was an outbreak of Severe Acute Respiratory Syndrome (SARS) in Taiwan. It has been reported that SARS had an impact on health care utilization.^[26]^ Therefore, data from 2003 were not used in this study.

### 2.3 Charlson comorbidity index

A score of 1 was added when the subjects had co-morbid myocardial infarction, congestive heart failure, peripheral vascular disease, cerebrovascular disease, dementia, chronic pulmonary disease, connective tissue disease, peptic ulcer disease, mild liver disease, or diabetes without end-organ damage. A score of 2 was added when the subjects had co-morbid hemiplegia, moderate or severe renal disease, diabetes with end-organ damage, tumor without metastases, leukemia, or lymphoma. A score of 3 was added when the subjects had co-morbid moderate or severe liver disease. A score of 6 was added when the subjects had co-morbid metastatic solid tumors or acquired immunodeficiency syndrome (AIDS). The total score was obtained by adding the relative weight of all co-morbidities. For each decade > 40 years of age, a score of 1 is added to the sum of the above-mentioned scores.^[27–29]^

### 2.4 Income state index

In Taiwan, health insurance premiums are calculated as a percentage of an individual's monthly salary. There were 7 income levels in this study: Level 0, no monthly income; Level 1, monthly salary income below Taiwan dollars (NT$) 17,280; Level 2, monthly salary income ranging from NT$ 17,281 to NT$ 22,800; Level 3, monthly salary income ranging from NT$ 22,801 to NT$ 28,800; Level 4, monthly salary ranging from NT$ 28,801 to NT$ 36,300; Level 5, monthly salary income ranging from NT$ 36,301 to NT$ 45,800; Level 6, monthly salary above NT$ 45,801 (Table 1).^[30]^

**Table 1:**
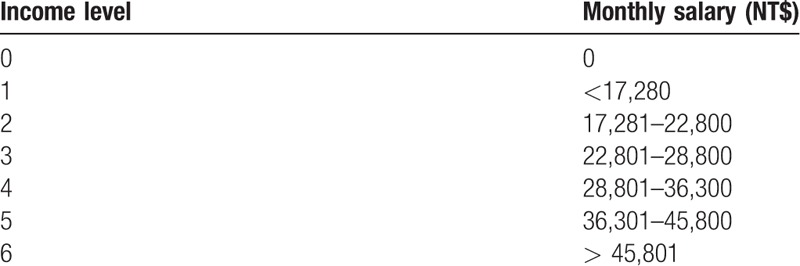
National health insurance premium level.

### 2.5 Ethics statement

The NHIRD encrypts patient personal information to protect privacy and provides researchers with anonymous identification numbers associated with relevant claims information, including sex, date of birth, medical services received, and prescriptions. Patient consent is not required to access the NHIRD. This study was approved by the Institutional Review Board (IRB) of China Medical University (CMU-REC-101-012). The IRB specifically waived the consent requirement.

### 2.6 Data availability statement

All data and related metadata were deposited in an appropriate public repository. The data on the study population that were obtained from the NHIRD (http://w3.nhri.org.tw/nhird//date_01.html) are maintained in the NHIRD (http://nhird.nhri.org.tw/). The NHRI is a nonprofit foundation established by the government.

### 2.7 Statistical analysis

Data are described as mean ± standard deviation (SD). The *t*-test was used to compare the differences in mean values of length of stay (LOS), diagnostic costs, drug costs, therapy costs, total costs, rate of revisiting the ED within 3 days, and 14-day re-admission rate before and after implementation of the global budget system.

Multilevel and mixed-effects models were employed to determine the impact of several independent variables on LOS, diagnostic costs, drug costs, therapy costs, total costs, the risk of revisiting the ED within 3 days, and the risk of being readmitted within 14 days after discharge. Mixed-effects modeling is basically regression analysis that takes into account 2 kinds of effect sizes: fixed effects (intercepts and slopes that describe the population as a whole) and random effects (intercepts and slopes that can vary across subgroups of the sample).^[31]^

There were 2 nested levels in this study: hospital accreditation levels and regional levels. There are three accreditation hospital levels in Taiwan: medical centers, regional hospitals, and local hospitals. Taiwan is divided into 6 geographical areas that include Taipei city, northern Taiwan, central Taiwan, southern Taiwan, Kaoshiung, and eastern Taiwan. The independent variables evaluated in this study included pre-post global budgeting, age, gender, income state index, Charlson Comorbidity Index, the 3 hospital levels, and the 6 geographic areas in Taiwan.

All statistical analyses were performed on a personal computer using the statistical package STATA for Windows (Version 11.0, College Station, TX). A *P-*value of .05 was considered to represent statistical significance.

## 3 Results

A total of 2810 patients with acute pancreatitis (1108 pre- and 1702 post-global budget) were recruited in this study. The mean ages of subjects before and after global budgeting were 47.75 ± 0.47 years and 50.83 ± 0.42 years, respectively. In the pre-budget group, 74% of the subjects were men and in the post-budget group, 75% of the subjects were men. The mean income state index before implementation of the global budgeting system was 1.79 ± 0.05 and that after adoption of the system was 1.74 ± 0.04. The mean Charlson Comorbidity Index before global budgeting was 0.75 ± 0.04 and that after introduction of the system was 1.00 ± 0.03. There were significant differences in age (*P* < .001), income state index (*P* = .04), and Charlson Comorbidity Index (*P* < .001) before and after the global budgeting system went into effect. There was no significant difference in male to female ratio before and after adoption of the global budget system *(P* = .10) (Table 2).

**Table 2:**
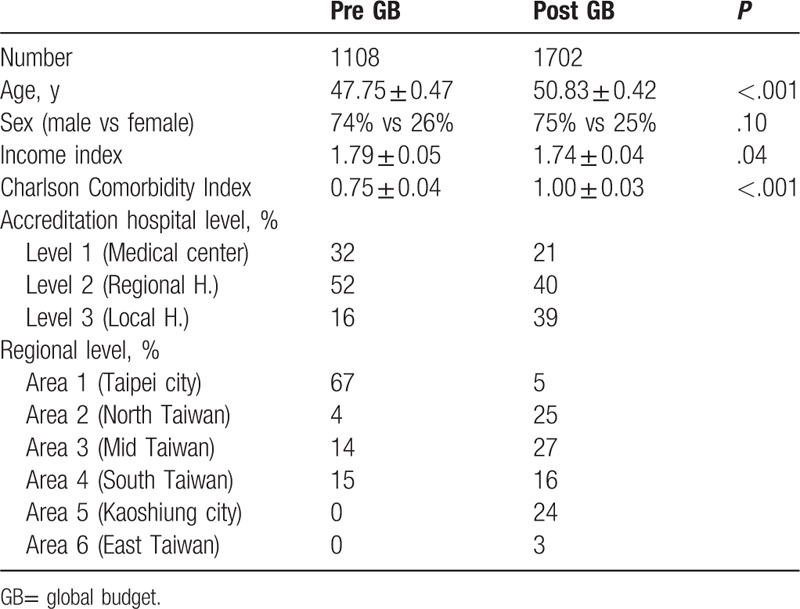
Demographic data of patients with acute pancreatitis pre and post implementation of GB.

The mean LOS before implementation of the global budget system was 7.34 ± 0.22 days and that after the system went into effect was 7.82 ± 0.22 days. The definitions of diagnostic cost, therapy costand drug cost were costs of laboratory diagnostic tests, treatment for acute pancreatitis, and medicines. The mean diagnostic costs (in NT$) before and after global budgeting were NT$ 2085.70 ± 64.42 and NT$ 2875.96 ± 86.96, respectively. The mean drug costs before and after implementation of the global budget system were NT$ 6363.78 ± 547.30 and NT$ 8723.39 ± 665.60, respectively. The mean therapy costs before and after the global budget were NT$ 1074.26 ± 137.10 and NT$ 3412.97 ± 320.40, respectively. The mean total costs before and after adoption of global budgeting were NT$ 28,290.66 ± 1576.32 and NT$ 42,341.83 ± 2285.23, respectively. The mean 3-day re-visit rate to the ED decreased from 9.9 ± 0.9% at baseline to 7.2 ± 0.6% after implementation of the global budget system. The mean 14-day re-admission rates before and after the global budget went into effect were 11.6 ± 1.0% and 7.9 ± 0.7%. There were significant differences in LOS, diagnostic costs, drug costs, therapy total costs, 3-day ED revisit rate, and 14-day re-admission rate before and after adoption of global budgeting (*P* = .001 for LOS and *P* < .001 for others) (Table 3).

**Table 3:**
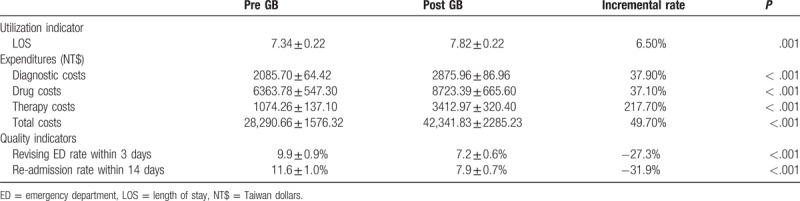
Compare the differences in mean values ofLOS, diagnostic costs, drug costs, therapy costs, total costs, rate of revisiting the ED within 3 days, and 14-day re-admission rate before and after implementation of the global budget system.

### 3.1 LOS

A mixed-effects Poisson model was used for clustered count data analysis.^[32]^ The Poisson regression model was fitted using option IRR (incidence-rate ratio) to obtain exponential estimates. Global budgeting did not have a significant impact on LOS (IRR = 0.99, *P* = .51). Male gender was associated with a significantly shorter LOS than female gender (IRR = 0.96, *P* = .016). In addition, LOS was positively correlated with age and Charlson Comorbidity Index (IRR = 1.003, *P* = .002; IRR = 1.11, *P* < .001). There was a significantly negative correlation between income state index and LOS (IRR = 0.97, *P* < .001). Compared with medical centers, there was no significant difference in LOS between regional hospitals or local hospitals (IRR = 1.04*, P* = .55; IRR = 1.05, *P* = .49). Compared with hospitals in Taipei city, LOS differed significantly among hospitals in northern Taiwan, central Taiwan, southern Taiwan, Kaoshiung city, and eastern Taiwan (IRR = 1.15, *P *= .14; β = 0.99, *P* = .91; β = 1.10, *P* = .32; β = 0.92, *P* = .38; β = 0.83, *P* = .14) (Table 4).

**Table 4:**
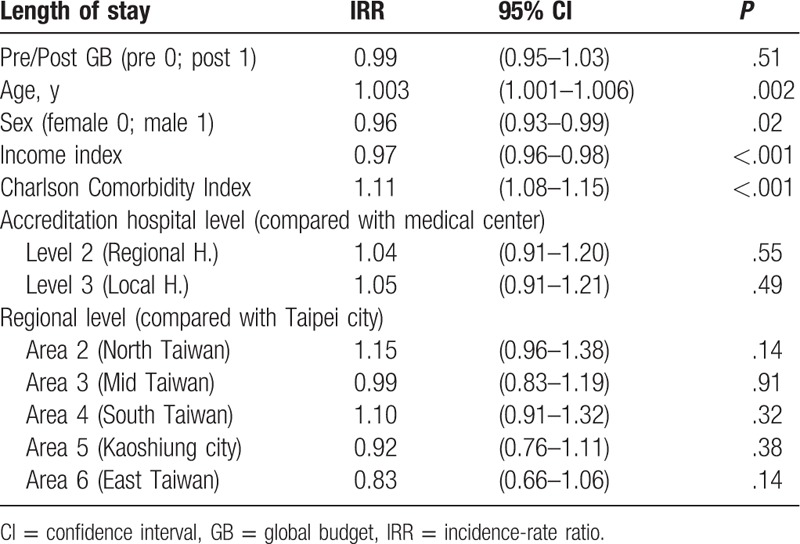
The impact of several independent variables on length of stay in patients with acute pancreatitis (mixed-effects Poisson model).

### 3.2 Diagnostic costs

A mixed-effects linear model was used for analysis. Because diagnostic costs were skewed to the right, data were converted to natural logarithm for statistical analysis. Global budgeting had a significantly positive impact on diagnostic costs (β = 0.19, *P* < .001). Male gender was associated with significantly lower diagnostic costs than female gender (β = −0.08, *P* = .01). There was no significant correlation between age and diagnostic costs (β = 0.003, *P* = .19). There was, however, a significantly negative correlation between income state index and diagnostic costs (β = −0.02, *P* = .01). Furthermore, there was a significantly positive correlation between Charlson Comorbidity Index and diagnostic costs (β = 0.08, *P* = .003). Compared with medical centers, there was no significant difference in diagnostic costs between regional hospitals and local hospitals (β = 0.001, *P* = .98; β = 0.03, *P* = .76). Compared with diagnostic costs incurred in hospitals in Taipei city, there were no significant differences in diagnostic costs among hospitals in northern Taiwan, central Taiwan, southern Taiwan, Kaoshiung city, and eastern Taiwan (β = 0.05, *P* = .43; β = 0.02, *P* = .80; β = 0.08, *P* = .24; β = −0.02, *P* = .82; β = −0.16, *P* = .20) (Table 5).

**Table 5:**
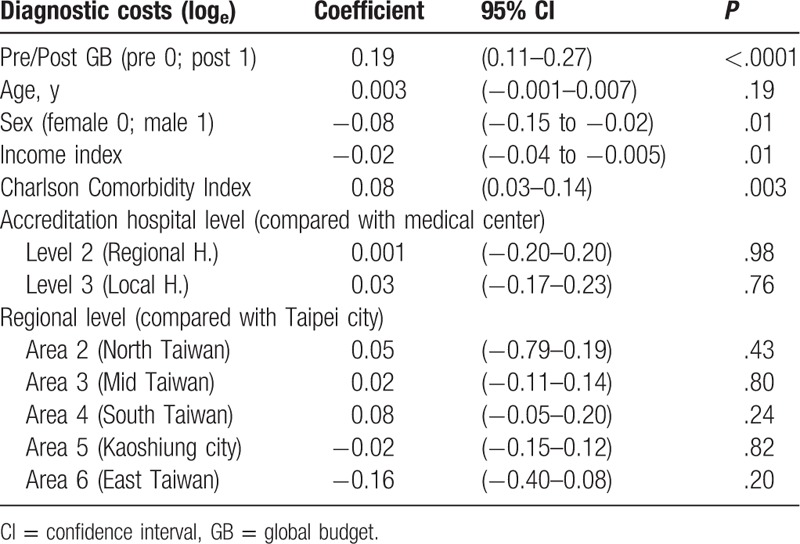
The impact of several independent variables on diagnostic costs, drug costs, therapy costs, and total costs in acute pancreatitis (mixed-effects linear model).

### 3.3 Drug costs

A mixed-effects linear model was used for analysis. Because drug costs were skewed to the right, data were converted to natural logarithm for statistical analysis. Global budgeting did not have a significant impact on drug costs (β = 0.03, *P* = .71). Male gender was associated with significantly lower drug costs than female gender (β = −0.13, *P* = .03). There was a significantly positive correlation between age and drug costs (β = 0.01, *P* = .003). There was, however, no significant correlation between income state index or Charlson Comorbidity Index and drug costs (β = −0.01, *P* = .42; β = 0.08, *P* = .14). Compared with medical centers, there was no significant difference in drug costs between regional hospitals and local hospitals (β = −0.04, *P* = .83; β = 0.003, *P* = .99). Compared with hospitals in Taipei city, there was no significant difference in drug costs among hospitals in northern Taiwan, central Taiwan, southern Taiwan, Kaoshiung city, and eastern Taiwan (β = −0.06, *P* = .57; β = −0.13, *P* = .18; β = 0.01, *P* = .90; β = −0.12, *P* = .27; β = −0.37, *P* = .09).

### 3.4 Therapy costs

A mixed-effects linear model was used for analysis. Because therapy costs were skewed to the right, data were converted to natural logarithm for statistical analysis. Global budgeting had a significantly positive impact on therapy costs (β = 0.38, *P* = .01). There was no significant correlation between gender, age, or income state index and therapy costs (β = 0.06, *P* = .63; β = 0.002, *P* = .76; β = 0.009, *P* = .81). There was, however, a significantly positive correlation between Charlson Comorbidity Index and therapy costs (β = 0.27, *P* = .006). Compared with medical centers, there was no significant difference in therapy costs between regional hospitals and local hospitals (β = −0.08, *P* = .82; β = 0.03, *P* = .93). Compared with hospitals in Taipei city, there were no significant differences in therapy costs among hospitals in northern Taiwan, central Taiwan, southern Taiwan, Kaoshiung city, and eastern Taiwan (β = −0.09, *P* = .74; β = −0.06, *P* = .79; β = 0.26, *P* = .29; β = 0.32, *P* = .25; β = −0.14, *P* = .75) (Table 6).

**Table 6:**
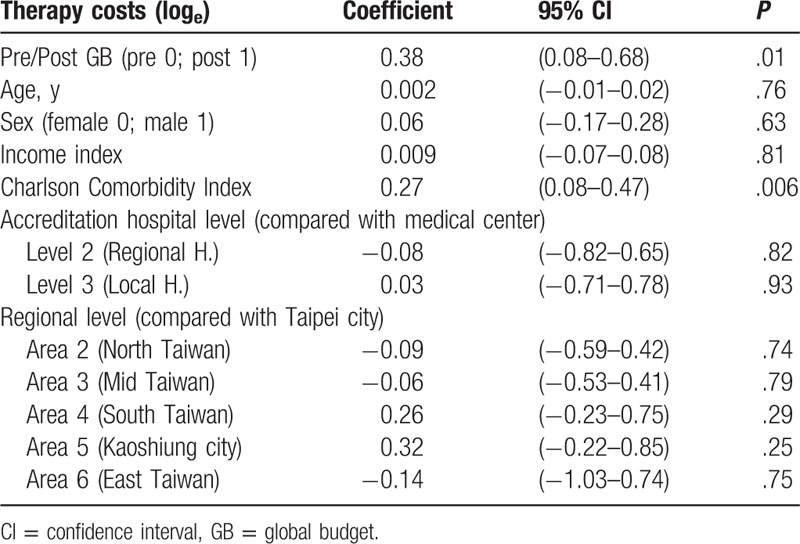
The impact of GB, age, sex, income index, Charlson Comorbidity Index, accreditation hospital level, and regional level on therapy costs (log_e_) using mixed-effects linear model.

### 3.5 Total costs

A mixed-effects linear model was used for analysis. Because total costs were skewed to the right, data were converted to natural logarithm for statistical analysis. Global budgeting had a significantly positive impact on total costs (β = 0.17, *P* = .02). Male gender was associated with significantly lower total costs (β = −0.15, *P* = .001). There was a significantly positive correlation between age and total costs (β = 0.008, *P* = .007). There was, however, no significant correlation between income state index and total costs (β = −0.02, *P* = .09). There was a significantly positive correlation between Charlson Comorbidity Index and total costs (β = 0.12, *P* = .002). Compared with medical centers, there was no significant difference in total costs between regional hospitals and local hospitals (β = −0.01, *P* = .94; β = 0.06, *P* = .66). Compared with hospitals in Taipei city, there was no significant difference in total costs among hospitals in northern Taiwan, central Taiwan, southern Taiwan, Kaoshiung city, or eastern Taiwan (β = 0.02, *P* = .85; β = 0.004, *P* = .96; β = 0.07, *P* = .38; β = −0.05, *P* = .58; β = −0.22, *P* = .17) (Table 7).

**Table 7:**
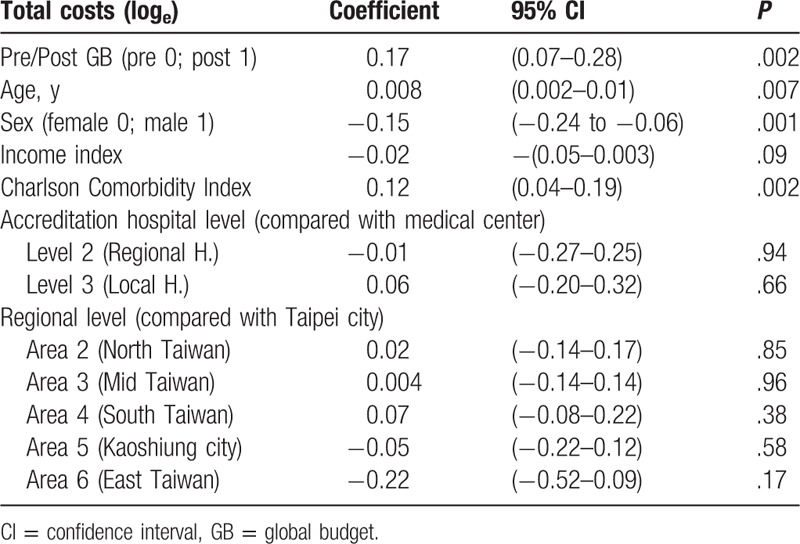
The impact of GB, age, sex, income index, Charlson Comorbidity Index, accreditation hospital level, and regional level on total costs (log_e_) using mixed-effects linear model.

### 3.6 Risk of revisiting the ED within 3 days

A generalized linear binary regression model was used for analysis. Global budgeting did not have a significant impact on the risk of revisiting the ED within 3 days (odds ratio, OR = 0.78; *P* = .21). In addition, there was no significant correlation between age, gender or income state index, and risk of revisiting the ED within 3 days (OR = 1.01, *P* = .27; OR = 0.90, *P* = .53; OR = 0.92, *P* = .12). There was, however, a significantly negative correlation between Charlson Comorbidity Index and risk of revisiting the ED (OR = 0.74, *P* = .04). Compared with medical centers, there was no significant difference in the risk of revisiting the ED within 3 days between regional hospitals and local hospitals (OR = 1.20, *P* = .27; OR = 0.74, *P* = .14). Compared with hospitals in Taipei city, there was no significant difference in the risk of revisiting the ED within 3 days among hospitals in northern Taiwan, central Taiwan, southern Taiwan, Kaoshiung city, or eastern Taiwan (OR = 0.78, *P* = .38; OR = 1.03, *P* = .86; OR = 1.26, *P* = .27; OR = 1.35, *P* = .27; OR = 4.21E-08, *P* = .99) (Table 8).

**Table 8:**
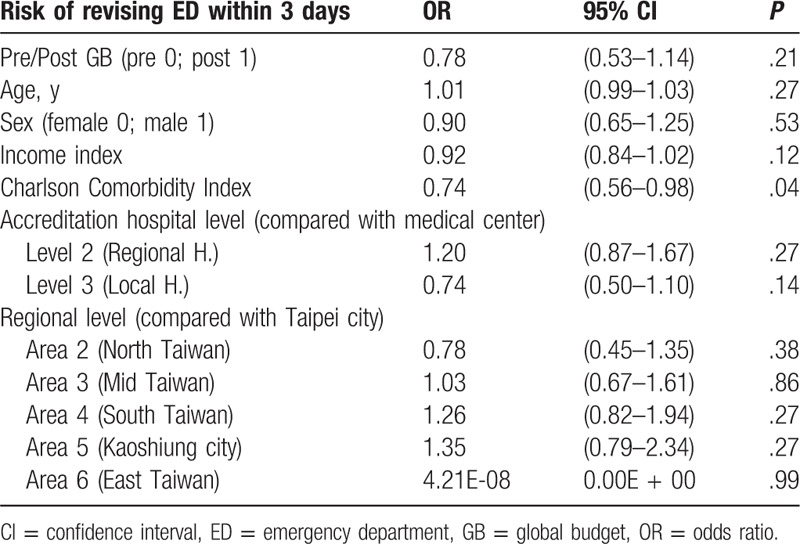
The impact of several independent variables on the risk of revisiting ED within 3 days and the risk of re-admission within 14 days among patients with acute pancreatitis (mixed-effects linear binary regression model).

### 3.7 Risk of readmission within 14 days

A generalized linear binary regression model was used for analysis. Global budgeting had a significantly negative impact on the risk of being readmitted within 14 days (OR = 0.65; *P* = .02). There were no significant correlations between age, gender, income state index, or Charlson Comorbidity Index and the risk of readmission within 14 days (OR = 1.02, *P* = .12; OR = 0.81, *P* = .17; OR = 0.97, *P* = .50; OR = 0.81, *P* = .14). In addition, there was no significant difference in the risk of being readmitted within 14 days between medical centers and regional hospitals (OR = 0.86, *P* = .33). However, patients treated in local hospitals were at significantly lower risk of readmission within 14 days than patients treated in medical centers (OR = 0.65, *P* = .02). Compared with hospitals in Taipei city, there was no significant difference in the risk of readmission within 14 days among hospitals in northern Taiwan, central Taiwan, southern Taiwan, Kaoshiung city, and eastern Taiwan (OR = 1.13, *P* = .63; OR = 1.33, *P* = .18; OR = 1.41, *P* = .10; OR = 1.22, *P* = .47; OR = 4347E-08, *P* = .99) (Table 9).

**Table 9:**
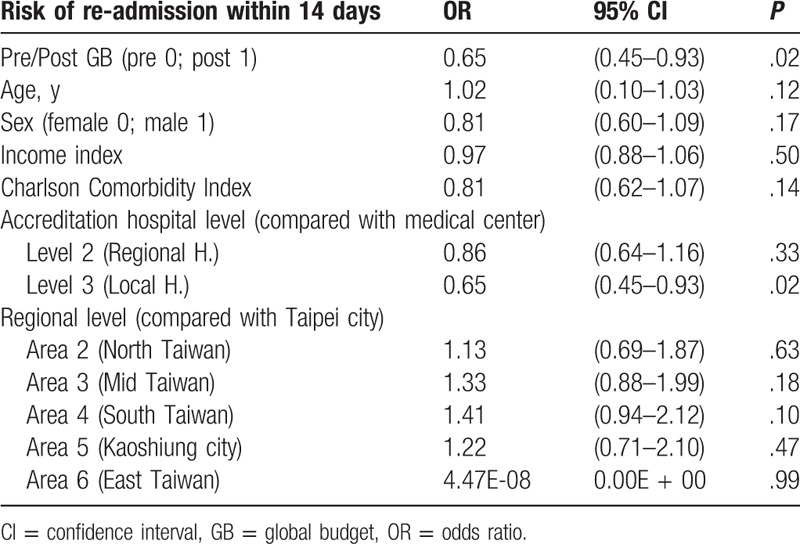
The impact of GB, age, sex, income index, Charlson Comorbidity Index, accreditation hospital level, and regional level on the risk of re-admission within 14 days using generalized linear binary regression model.

## 4 Discussion

The number of patients with acute pancreatitis increased by 53.6% after implementation of global budgeting. Alcohol is one of the leading causes of acute pancreatitis. The prevalence of alcoholism increased in Taiwan. This might partially explain why the number of cases of pancreatitis increased after adoption of the global budgeting system.^[33]^ The LOS was significantly longer after global budgeting took effect. Aging and more severe forms of morbidity may have contributed to longer hospital stays in patients with acute pancreatitis.

Diagnostic costs, drug costs, therapy costs, and total costs all significantly increased after implementation of the global budget system. This represents the increases of 37.1% to 217.7%, significantly exceeding Taiwan's 2.2% growth in consumer price index (CPI) for the same period (Appendix 1). Possible reasons for the incremental increases in health care expenditures during the study period include longer LOS, older age, and the presence of more comorbidities in the study population. Another possible cause is that the in an expenditure cap system, the budget is fixed ex ante but the reimbursement prices are determined ex poste. Once the services have been provided, the prices of those services are adjusted so that the fixed budget will cover them. In this type of situation, providers are in a situation similar to Cournot competitors, having to make supply decisions before knowing the price they will receive for the service, because the price of the service will be determined by the summed supply decisions of all the providers. Each provider will have an incentive to increase their supply and result in a rise in the total costs post global budgeting.

Possible cause of significant incremental mean costs is the extreme values of costs, because the mean values will be affected by extreme values. The incremental rates in mean diagnostic costs, drug costs, therapy costs and total costs were 0.38, 0.37, 2.18, and 0.50. The different rates in median diagnostic costs, drug costs, therapy costs and total costs were 0.23, −0.08, 0, and 0.21. Differences in median costs were much smaller than the mean costs. There was no difference in median therapy costs before and after global budgeting. The median drug cost after global budgeting was even lower than that before global budgeting. There was no significant difference in health care expenditures for acute pancreatitis between hospitals with different accreditation levels or between different geographic areas in Taiwan.

The risk of revisiting the ED within 3 days and the risk of being readmitted within 14 days were significantly lower after global budgeting went into effect. Those findings indicate that the quality of care of patients with acute pancreatitis improved after adoption of the global budgeting system. Improvements in care may be attributed to the use of more effective antibiotics, advances in standard operative procedures, and better-trained medical providers. After adjusting for other covariates, there was a significantly negative correlation between global budgeting and the risk of readmission within 14 days. The impact of global budgeting on the risk of revisiting the ED within 3 days, however, was not significant. Neither hospital accreditation level nor geographic area was associated with the risk of revisiting the ED within 3 days. Nevertheless, patients treated at local hospitals were at significantly lower risk of readmission within 14 days than patients treated at medical centers. Risk factors for early re-admission in patients with acute pancreatitis include moderate to heavy alcohol use, persistent gastrointestinal symptoms (nausea, vomiting, or diarrhea), and diet at the time of discharge.^[34]^ Those patient characteristics were not available from the database; it is, therefore, not clear whether those factors played a role in early readmission in this study. The reasons why patients treated in local hospitals were at a lower risk of readmission within 14 days than patients in medical centers is also not clear. Whether patients with acute pancreatitis in local hospitals received better quality care than patients in medical centers or whether patients in medical centers had more risk factors for early re-admission needs to be investigated.

In Taiwan's market-driven health-care system, 65% of hospitals are private (department of Health, 2001). Sixty-three percent of physicians are salaried employees of hospitals, some receiving bonus payments based on productivity. In response to an expenditure cap, physicians increased their activity levels including do more tests, provide more complex and high-priced procedures which make diagnostic costs, therapy costs, and total costs significantly increased after global budgeting. The major treatment for acute pancreatitis includes supportive care, fluid resuscitation, transfer to intensive care unit and enteral feeding. Since routine use of prophylactic antibiotics in patients with severe acute pancreatitis and/or sterile necrosis is not recommended; drug costs after global budget were not affected significantly. It has been reported that the launch of global budgets leads to an increase in the quantity of medical services supply to maximize profits. In order to maximize revenue, the medical providers in Taiwan present incentives to increase medical care utilization and result in significantly longer length of stay after global budgeting. Market competition between hospitals continues to increase in the health-care industry. For survival, hospitals must focus on quality of care. Furthermore, if the hospital has the plans to improve its service quality, there is more room for a hospital to negotiate the expenditure cap with the BNHI. Thus quality of care significantly improved after global budgeting in Taiwan.

This study was subject to limitations. First, the NHIRD provides no detailed information on patients regarding factors such as their lifestyle, habits, body mass index, physical activity, socioeconomic status, or family history; all of which are possible confounding factors in this study. Second, the evidence derived from a cohort study is generally of lower methodological quality than that from randomized trials because a cohort study is subject to many biases related to the necessary adjustments for confounding factors. Despite the meticulous design of this study and its adequate control of confounding factors, biases could remain because of possibly unmeasured or unknown confounding factors. Third, the registries in the NHI claims are primarily used for administrative billing and are not verified for scientific purposes. Because of the anonymity of the identification numbers, obtaining additional information by directly contacting the patients was not possible. The accuracy of medical coding in the claims data may affect the data validity. However, the data on the diagnoses in the NHIRD are highly reliable. The insurance system has mechanisms to monitor the insurance claims.

## 5 Conclusion

The global budget system was associated with significantly longer length of stay, higher health care expenditures, and better quality of care in patients treated for acute pancreatitis.

## Author contributions

**Provision of study materials**: Ya-Lin Ko, Jyun-Wei Wang, Hui-Mei Hsu, Chia-Hung Kao, Chun-Yi Lin.

**Data collection**: Ya-Lin Ko, Jyun-Wei Wang, Hui-Mei Hsu, Chia-Hung Kao, Chun-Yi Lin.

**Data analysis and interpretation**: Ya-Lin Ko, Jyun-Wei Wang, Hui-Mei Hsu, Chia-Hung Kao, Chun-Yi Lin.

**Final approval**: Ya-Lin Ko, Jyun-Wei Wang, Hui-Mei Hsu, Chia-Hung Kao, Chun-Yi Lin.

**Conceptualization:** Chun-Yi Lin.

**Data curation:** Chun-Yi Lin.

**Formal analysis:** Ya-Lin Ko, Jyun-Wei Wang, Chia-Hung Kao, Chun-Yi Lin.

**Funding acquisition:** Chun-Yi Lin.

**Investigation:** Chun-Yi Lin.

**Methodology:** Chun-Yi Lin.

**Project administration:** Chun-Yi Lin.

**Resources:** Chun-Yi Lin.

**Writing – original draft:** Chun-Yi Lin.

**Writing – review & editing:** Hui-Mei Hsu, Chun-Yi Lin.
